# Construction and validation of a novel signature based on epithelial-mesenchymal transition–related genes to predict prognosis and immunotherapy response in hepatocellular carcinoma by comprehensive analysis of the tumor microenvironment

**DOI:** 10.1007/s10142-022-00933-w

**Published:** 2022-12-20

**Authors:** Biao Gao, Yafei Wang, Shichun Lu

**Affiliations:** 1 School of Medicine, Nankai University, Tianjin, 300300 China; 2 Faculty of Hepato-Pancreato-Biliary Surgery, Chinese PLA General Hospital, Beijing, 10058 China; 3 Institute of Hepatobiliary Surgery of Chinese PLA, Beijing, 10058 China; 4 Key Laboratory of Digital Hepetobiliary Surgery PLA, Beijing, 10058 China

**Keywords:** Hepatocellular carcinoma, EMT, Immunotherapy, TMB, MSI

## Abstract

Immunotherapy has yielded encouraging results in the treatment of advanced hepatocellular carcinoma (HCC). However, the relationship between epithelial-mesenchymal transition (EMT) and immunotherapy for HCC has not been adequately explained. In this study, we comprehensively analyzed a bulk RNA sequence dataset of 365 HCC patients in The Cancer Genome Atlas (TCGA) dataset. Subsequently, we constructed a prognostic signature based on 6 EMT-related genes and divided 365 HCC patients into high- and low-risk groups. The predictive efficacy of the signature was well validated in different clinical subgroups and in two independent external datasets. We further explored the relationship between prognostic signature and immunotherapy response in terms of immune cell infiltration, somatic mutations, tumor mutation burden (TMB), microsatellite instability (MSI), immune checkpoint–associated gene expression, single-nucleotide variants (SNV) neoantigens, cancer testicular antigens (CTA) scores, and tumor immune dysfunction and exclusion (TIDE) scores. We validated the predictive efficacy of prognostic signature for immunotherapy response using external independent immunotherapy data. Real-time quantitative polymerase chain reaction (qRT-PCR) was used to validate EMT-related gene overexpression in HCC tissue samples. Prognostic signature was an independent risk factor affecting the prognosis of HCC patients and has shown superiority in predicting patient survival compared to other clinical factors. Compared with the low-risk group, the proportion of Activated_CD4_T_cell, Type_2_T_helper_cel, and macrophages were higher in the tumor microenvironment of HCC patients in the high-risk group, while the Activated_CD8_T_cell and CD56bright_natural_killer_cell proportions were lower. The prognostic signature was positively correlated with TMB scores, MSI scores, SNV neoantigens scores, expression levels of immune checkpoint–related genes, and TIDE scores, and patients in the high-risk group were more suitable for immunotherapy. qRT-PCR confirms overexpression of 6 EMT-related genes in HCC tissues for the construction of prognostic signature. Our novel prognostic signature can effectively predict the prognosis and immunotherapy response of HCC patients. In the future, it will be an effective tool for physicians to screen suitable immunotherapy populations and improve response rates and overall survival (OS).

**Supplementary Information:**

The online version contains supplementary material available at 10.1007/s10142-022-00933-w.

## Introduction

Primary liver cancer is one of the most common malignant tumors, among which hepatocellular carcinoma (HCC) accounts for 85–90% of primary liver cancer (Zhou et al. 2018). HCC starts insidiously; 39.0–53.6% of patients are already in an advanced stage at the time of diagnosis, losing the opportunity for radical surgery (Li et al. 2021). HCC is highly heterogeneous, with approximately 80–90% of cases developing from cirrhosis and chronic liver disease (Akinyemiju et al. 2017; Chidambaranathan-Reghupaty et al. 2021), as well as a high malignancy and recurrence rate (EASL-EORTC clinical practice guidelines 2012). The tumor microenvironment (TME) plays a critical role in the development and treatment of HCC (Kurebayashi et al. 2018), as it is where cancer cells interact with multiple immune components to create a suppressive immune microenvironment that promotes immune escape, proliferation, invasion, and metastasis of cancer cells, as well as mediates drug resistance (Pinato et al. 2020). Immune checkpoint inhibitor–related therapy reactivate tumor-specific immunity and normalize the function of immunocompetent cells (Johnston and Khakoo 2019). However, immunotherapy for HCC is only 15–25% effective in real-world clinical trials (Zhang et al. 2018; Pinter et al. 2021), making it critical to identify potential biomarkers to identify populations that may benefit from immunotherapy for clinician decision-making and treatment outcomes for patients with liver cancer.

In this study, we performed a comprehensive analysis of bulk RNA sequence data and somatic mutation data from 365 HCC patients in TCGA, and we constructed a prognostic signature based on the expression of 6 EMT-related genes. To further explore the relationship between prognostic signature and immunotherapy, we comprehensively profiled the composition of immune cells in the tumor microenvironment of each patient. TMB, MSI, CTA score, SNV neoantigen score, mRNA expression levels of immune checkpoint–related genes, and TIDE score were used to evaluate the efficacy of immunotherapy in different risk groups. Interestingly, based on the prognostic signature, we succeeded in screening a group of HCC patients that may respond strongly to immunotherapy. Therefore, in the future, the prognostic signature we constructed may provide an important reference value for clinicians to screen the immunotherapy population.

## Materials and methods

### Data and clinical samples

We used TCGA (https://portal.gdc.cancer.gov/) (Colaprico et al. 2016) to collect gene expression data and clinical information with complete prognosis for 424 HCC patients, comprising 365 tumor samples and 59 normal tissue samples, as the training set. The clinical features of the 365 HCC patients included in the training set are summarized in Table 1. Additionally, we used the Epithelial-Mesenchymal Transition Gene Database (http://dbemt.bioinfo-minzhao.org/) to get the EMT-related gene set. To verify the validity of the prognostic signature, we collected gene expression data and related clinical data of 231 HCC patients from the ICGC (https://dcc.icgc.org/) database. Meanwhile, we downloaded gene expression data and relevant clinical data of 220 HCC patients(GSE14520) from the GEO database. Immunotherapy data of tumor patients were obtained from GSE91061.

**Table 1:** Clinical characteristics of HCC patients in TCGA

Characteristics	Samples (*n* = 365)	Percentage (%)
Gender		
Female	120	32.9
Male	245	67.1
Age		
≥ 60	200	54.8
< 60	165	45.2
Stage		
Stage I	180	49.3
Stage II	91	25.0
Stage III	81	22.2
Stage IV	13	3.6
M		
M0	263	72.1
MX	102	28.0
N		
N0	248	67.9
N1	5	1.4
NX	112	30.7
Grade		
G1 G2 G3 G4	57 176 119 13	15.6 48.2 32.6 3.7

### Identification of differentially expressed EMT-related genes

We used the “limma” package of the R software (version 4.1.2) to analyze the differences between 365 tumors and 59 normal samples. The screening criteria were *p* < 0.05, log (FC) > 1, or log (FC) < 1 to obtain the differentially expressed genes (DEG) in the TCGA dataset (Robinson et al. 2010). We took the intersection of differentially expressed genes in HCC tumor tissues and normal tissues with EMT-related genes to obtain the differentially expressed EMT-associated genes.

### Functional enrichment analysis and gene set enrichment analysis

To investigate the molecular mechanisms underlying differential EMT, we used the “clusterProfiler” package of the R software to perform functional enrichment analysis on differentially expressed genes using GO (Gene ontology) and KEGG (Kyoto Encyclopedia of Genes and Genomes) annotations (Yu et al. 2012), with *p* < 0.05 considered statistically significant. Gene set enrichment analysis by the GSEA software calculates the enrichment scores of low- and high-risk group samples. The gene expression profile of the two risk groups was used to evaluate the related pathways and molecular mechanisms, and the reference gene sets “c2.cp.kegg.v7.4.symbols” were downloaded from the molecular signature database (https://www.gsea-msigdb.org/gsea/msigdb).

### Construction and validation of prognostic signature based on EMT-related genes

On 365 patients with HCC in the training set, we used COX univariate analysis to find genes with prognostic significance (*p* < 0.05). To eliminate overfitting, a least absolute shrinkage and selection operator (LASSO) Cox regression analysis was used in conjunction with the “glmnet” package. Finally, we utilized Cox multivariate analysis on the screened variables to find independent prognostic risk factors and construct an EMT prognostic signature. The risk score was calculated following the formula: risk score = ∑(Expi × Coefi). Coefi and Expi denote the risk coefficient and gene expression, respectively. Following that, all patients were classified as high-risk group and low-risk group based on their median risk score. The prediction performance of the prognostic signature in the training set was determined using Kaplan–Meier, log-rank tests and time-dependent receiver operating characteristic curves (ROC). Similarly, we calculated the risk score for each patient in the ICGC data set using the same formula and classified HCC patients into high-risk and low-risk groups based on the median risk score. In the GSE14520 validation set, we calculated the risk score for each patient using the same formula and classified HCC patients into high-risk and low-risk groups using the best cut-off value. Kaplan–Meier survival curves were used to compare the difference in survival between the two groups. ROC curves were used to validate the predictive efficacy of risk scores on the prognosis of HCC patients. To further validate the robustness of the prognostic model, we included different clinical characteristics in the study and validated the prognostic signature between different clinical subgroups. ROC curves were used to compare the superiority with other EMT prognostic signatures.

### Immune cell infiltration analysis and GSVA

CIBERSORT is a deconvolution technique that utilizes RNA-Seq data to determine the makeup of immune cells. We calculated the percentage of immune cell infiltration in 365 HCC patients using the CIBERSORT algorithm to compare the difference between infiltrated immune cells between the high-risk and low-risk groups. Gene set variation analysis (GSVA), a gene set enrichment method that estimates variation of pathway activity over a sample population in a nonparametric and unsupervised manner, showed an increased ability to deal with molecular profiling experiments compared to other methods (Hänzelmann et al. 2013). We assessed differences in the abundance of 28 immune cell gene sets between high- and low-risk groups using the GSVA function of R software.

### Assessment of stromal and immune scores

The ESTIMATE (Estimation of Stromal and Immune Cells in Malignant Tissue Using Expression Data) algorithm (via the estimation package in R) was used to quantify immune, stromal, and estimated scores, as well as tumor purity, using a gene expression matrix (Xu et al. 2021a). We calculated stromal score, immune score, ESTIMATE score, and tumor purity score for 365 HCC samples, and subsequently, we compared their differences between high and low risk groups.

### Mutation analysis and microsatellite instability

To further compare the differences in tumor cell mutant genes and tumor mutation burden(TMB) between high-risk and low-risk groups, the TCGA liver cancer mutation database (https://portal.gdc.cancer.gov/) was used to gather mutation data. We used the “maftools” package of the R software to conduct additional analysis on the mutation data and to calculate the tumor mutation burden (TMB) for each patient using the formula: TMB = (total mutations/total number of tests) × 10^6^. Microsatellite instability, a phenomenon of microsatellite sequence length change due to insertion or deletion of repetitive units during DNA replication, is often caused by defects in mismatch repair function. Clinical applications of microsatellite instability assays include a primary screening tool for Lynch syndrome, a prognostic factor for stage II colorectal cancer, and a predictor of immunotherapy efficacy in advanced solid tumors (Roudko et al. 2021; Rizzo et al. 2021). To explore the relationship between prognostic signature and immunotherapy, we compared the differences between high-risk and low-risk groups in MSI.

### Prediction of immunotherapy response

First, to compare the differences between the high-risk and low-risk groups in terms of immunotherapy response, I extracted the mRNA expression of immune checkpoint–related genes (PD-L1, PD-1, TIGIT, TIM-3, PD-L2, and CTLA4) commonly used in HCC. Subsequently, we examined the differences in the expression of these immune checkpoint–associated genes between the two groups using the Wilcoxon test. Tumor neoantigens are new antigens produced by tumor cells, and a growing number of findings show that immunotherapy enhances the immune system to recognize neoantigens on the surface of tumor cells to kill them, so the number of neoantigens in tumor cells correlates with the efficacy of PD-1/PD-L1 antibodies (Newell et al. 2022). The cancer testicular antigens (CTA) score is used to evaluate tumor immunogenicity, which indirectly reflects the strength of the immunotherapy response. To further evaluate the relationship between the prognostic model and immunotherapy response, we further compared the differences between different risk groups in terms of SNV neoantigen scores and CTA scores. In addition, the tumor immune dysfunction and exclusion algorithm was used to predict HCC patients’ immunotherapy response. To fully assess the differences between the two groups in terms of immunotherapy response, we further compared the differences between the two groups in terms of TIDE scores and dysfunction scores. We then calculated the risk score for each patient from the external independent immunotherapy data using the same formula and classified all patients into high- and low-risk groups based on the median value of the risk score. We further compared the difference in the proportion of CR/PR (CR: complete response; PR: partial response) between the two groups.

### Verification of mRNA expression level of six EMT-related genes by qRT-PCR in HCC

In order to verify the expression level of 6 EMT-related genes in HCC tissue samples, we collected tumor tissue samples and tumor-adjacent tissue samples from 10 HCC patients. We further compared the expression differences of six EMT-related genes in HCC tissue samples and adjacent tissues using qRT-PCR.

### Statistical tests

The R software was used to carry out every statistical analysis (Version 4.1.2). For significance labeling, the Wilcoxon test was used to compare the differences in variation between two groups of samples, while the Kruskal–Wallis test was utilized to compare the differences in variation between three or more groups of samples. To reduce the number of candidate genes and improve the accuracy of the EMT risk score, the univariate Cox analysis and the LASSO method were utilized. Two-tailed *P*-value less than 0.05 was set as the significantly different criteria.

## Results

### Identification of differential EMT-related genes

**Fig. 1:**
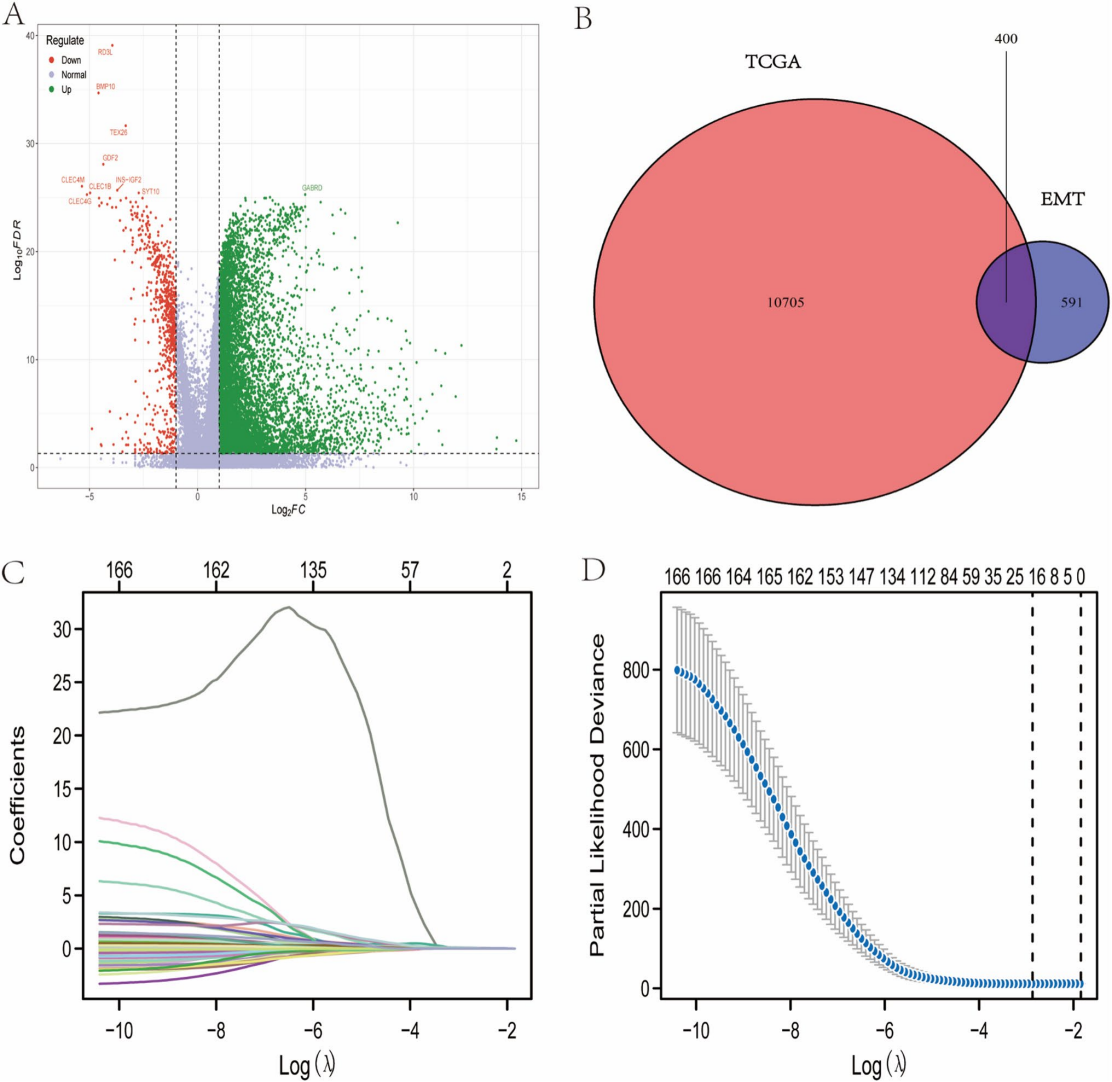
Identification of differentially expressed EMT-related genes. (**A**) Volcanic map shows the differentially expressed genes in HCC tissues and adjacent tissues. (**B**) Venn diagram shows the acquisition of differentially expressed EMT-related genes. (**C**) The coefficients of genes calculated by multivariate Cox regression using LASSO. (**D**) The partial likelihood deviance of genes

A total of 10,705 DEGs were discovered, consisting of 10,059 upregulated and 646 downregulated genes, using an adjusted *P*-value < 0.05 and a |log2 (fold change) of |> 1 (Fig. 1A). Following that, 400 DEG-EMTs were extracted (Fig. 1B). For the 400 differentially expressed EMT-related genes, we performed GO enrichment analysis. The GO enrichment analysis revealed that the most enriched biological process (BP) was mesenchyme development and epithelial tube morphogenesis; the most enriched cellular component (CC) was focal adhesion and cell-substrate junction, and the DNA-binding transcription activator activity and DNA-binding transcription activator activity, and RNA polymerase II-specific were significantly enriched in terms of molecular function (MF) (Fig. S1A).

### Construction and validation of EMT-related gene prognostic signature

**Fig. 2:**
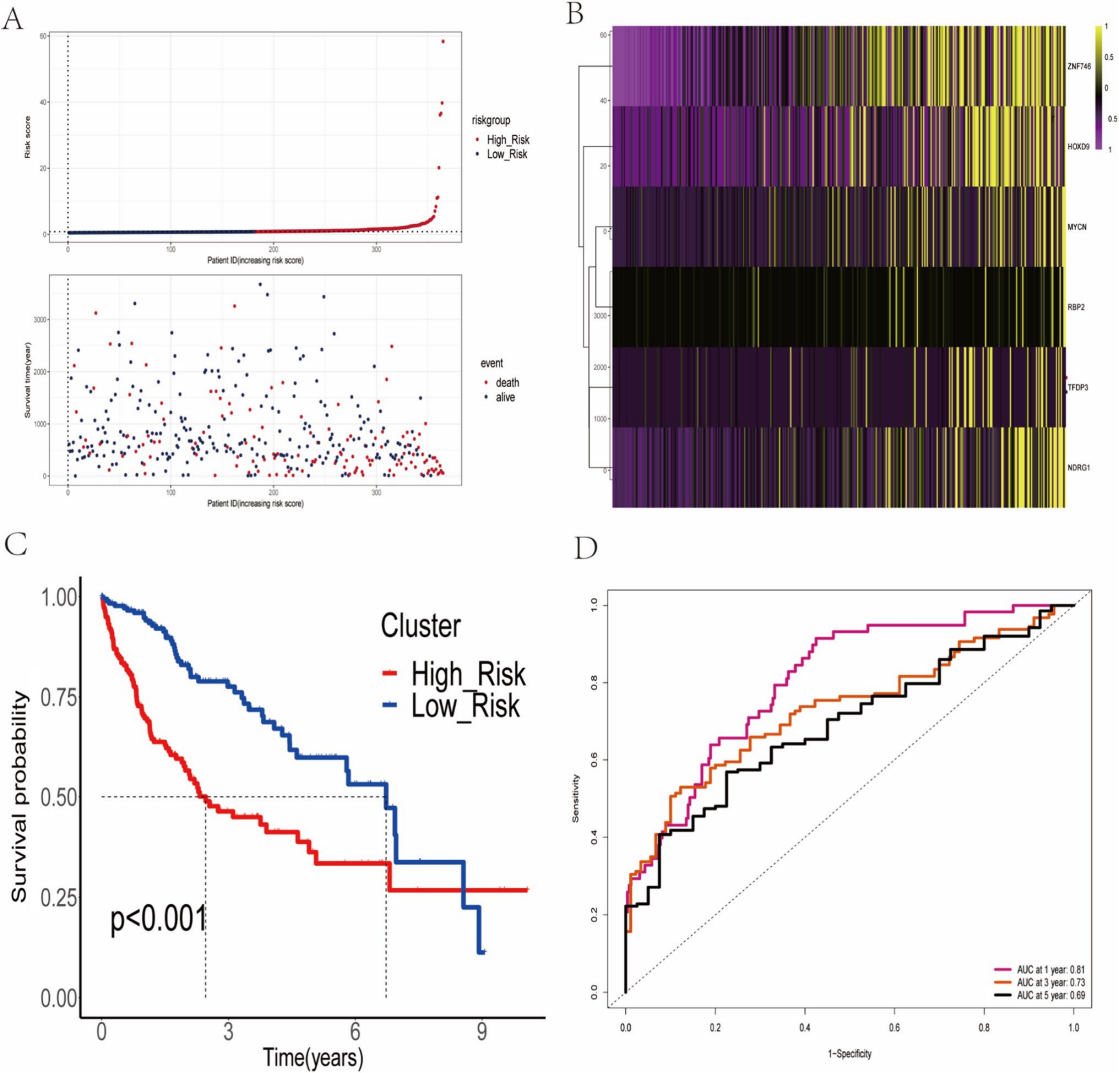
Construction of a prognostic signature for EMT-related genes. (**A**) The distribution of risk score and survival status. (**B**) Heatmap showed the expression characteristics of the identified 6 EMT-related genes. (**C**) Kaplan–Meier curves for survival analysis comparing overall survival of HCC patients between the high- and low-risk groups. (**D**) ROC curves of the prognostic signature for predicting the risk of death at 1, 3, and 5 years

We combined the gene expression data and clinical data from 365 HCC samples in the training set. We identified 166 prognosis-related genes using Cox univariate regression analysis; subsequently, we performed LASSO regression analysis on 166 genes and screened 16 model best variables (Fig. 1C–D). We further performed Cox multivariate analysis on the 16 variables and screened 6 independent risk factors affecting the prognosis of HCC patients used to construct a prognostic signature (Figure S1B). Six prognostic genes were identified in the training set used to build the prognostic signature (MYCN, ZNF746, TFDP3, NDRG1, HOXD9, and RBP2). The following formula was used to calculate prognostic risk scores: RiskScore = (0.103805 × MYCN gene expression) + (0.316454 × ZNF746 gene expression) + (0.074982 × TFDP3 gene expression) + (0.007409 × NDRG1 gene expression) + (0.132607 × HOXD9 gene expression) + (0.018091 × RBP2 gene expression). Based on the median risk score, we divided 365 HCC patients into high-risk group and low-risk group. Figure 2A depicts the distribution of risk scores and survival status, indicating that there are more deaths in the high-risk group. The heatmap demonstrates a positive correlation between the expression levels of six EMT-related genes and risk scores (Fig. 2B). Kaplan–Meier survival curve analysis showed that patients in the high-risk group had significantly worse survival than those in the low-risk group (*p* < 0.0001) (Fig. 2C); the area under the curve (AUC) for this risk signature was 0.785 at 1 year, 0.698 at 3 years, and 0.656 at 5 years (Fig. 2D).

### Validation of the prognostic signature in different clinical subgroups

We first explored the relationship between risk score and clinical characteristics, including age, gender, tumor grade, N stage, M stage, tumor stage (stage), microvascular invasion (MVI), and survival status. Interestingly, we found significant correlations between risk scores and tumor grade, MVI, stage, and survival status (*p* < 0.05). Patients with higher tumor grade and stage also had higher risk scores. Similarly, risk scores were significantly higher in patients with positive MVI and death (Figure S2). The superior predictive efficacy of the prognostic signature was initially validated in different clinical subgroups in the training set. High-risk scores were significantly associated with poorer prognosis in the young (age < 60) (*p* < 0.012), old (age > 60) (*p* < 0.001), female (*p* = 0.021), male (*p* < 0.001), low tumor grade (G1 + G2) (*p* < 0.001), high tumor grade (G3 + G4) (*p* = 0.006), MVI negative (*p* = 0.002), MVI positive (*p* = 0.001), early stage (Stage I + Stage II) (*p* < 0.001), and late stage (Stage III + Stage IV) (*p* = 0.002) (Figure S3-S4). These results preliminarily confirm that our prognostic model constructed based on EMT-related genes is equally applicable to different clinical subgroups.

### External dataset validates the predictive performance of the prognostic signature

We validated the robustness and validity of the prognostic model using 2 independent external datasets, and the clinical characteristics of the 2 independent external datasets are shown in Supplement Table 1. We calculated the risk score for each patient in the validation set using the same formula and divided HCC patients into high- and low-risk groups based on the median or best cutoff value of the risk score. The Kaplan–Meier analysis revealed that the overall survival time of high-risk patients was considerably shorter than that of low-risk patients in two external cohorts: GSE14250 (Figure S5A; HR = 3.011, 95% CI: 1.218–7.441, *p* = 0.017) and ICGC (Figure S5B; HR = 2.125,95%CI: 1.142–3.956, *p* = 0.0174). In both independent external datasets, the ROC curves confirmed that the risk score had good predictive efficacy for 1-, 3-, and 5-year survival in HCC patients (Figure S5C-S5D). In addition, we evaluated the cumulative predictive value of train sets (TCGA) and two validation sets for the prognosis of HCC patients by conducting a prognostic meta-analysis employing the random effects model R procedure meta. The results of the meta-analysis indicated that the prognostic signature was a highly accurate predictor for the prognosis in HCC patients (Figure S5E; HR = 2.44, 95% CI = 1.82–3.27).

### Identification of the prognostic signature as an independent prognostic factor

**Fig. 3:**
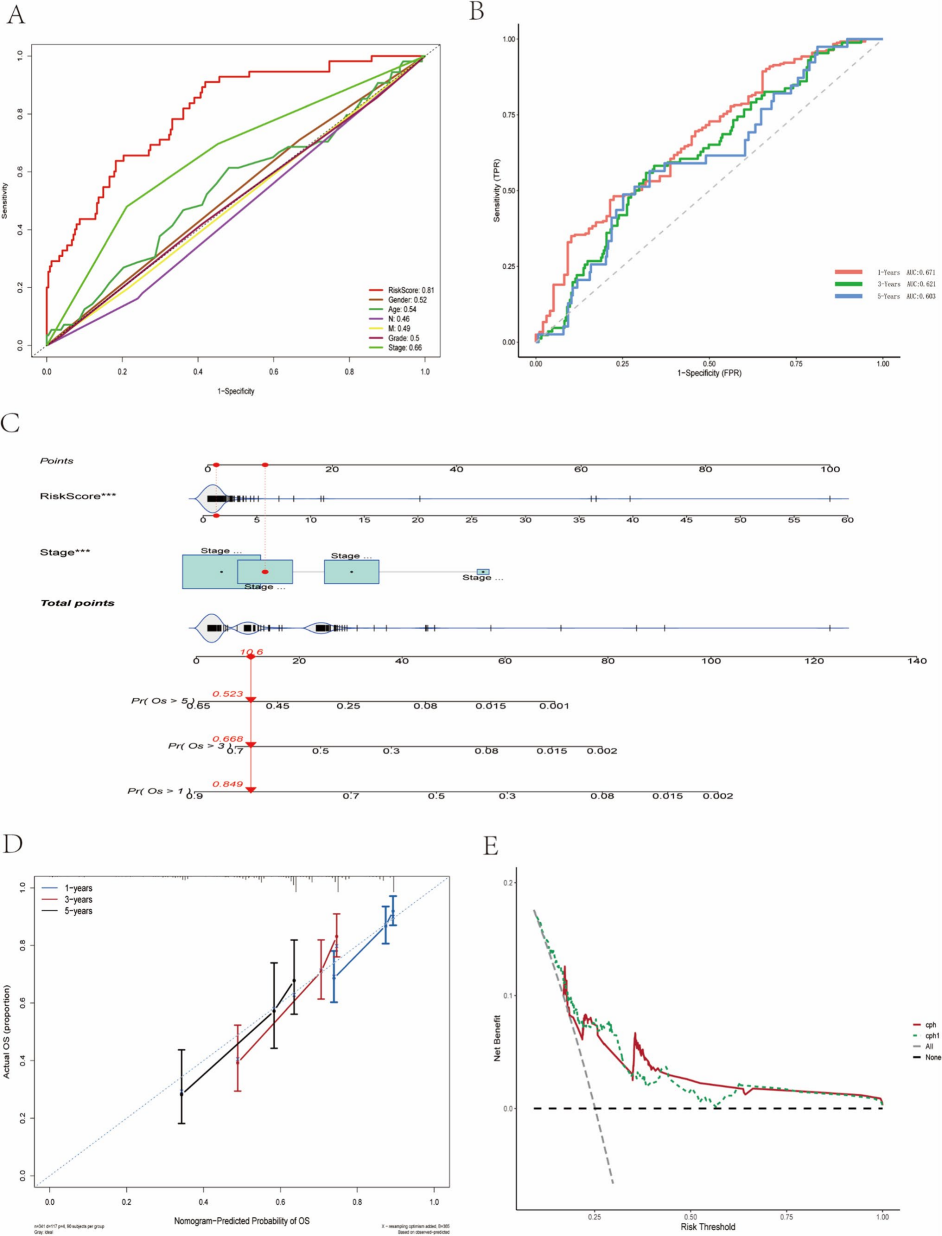
Nomogram construction and validation in TCGA LIHC dataset. (**A**) ROC curves showing the comparative results of risk scores and other clinical factors for prognostic prediction. (**B**) ROC curves showing the results of nomogram for predicting 1-, 3-, and 5-year survival in HCC patients. (**C**) Nomogram constructed from Stage and risk score. (**D**) Calibration curves show strong predictive power of nomogram for 1-, 3-, and 5-year survival in HCC patients. (**E**) Decision curves show that the nomogram model outperforms other models

We performed Cox univariate and multivariate analyses on the training set patients to assess whether risk scores could independently predict the prognosis of HCC patients. Cox multivariate analysis showed that stage and risk scores were associated with prognosis (*p* < 0.05). The results of multivariate Cox analysis showed that stage and risk score were associated with prognosis (*p* < 0.05), and the results indicated that risk score and stage could be used as independent predictors (Table 2). The area under the ROC curve for the risk score was larger than that for other clinical characteristics, further indicating that the risk score has better predictive performance than other clinical characteristics for the prognosis of HCC patients (Fig. 3A). To be able to predict the prognosis of HCC patients more accurately, we combined stage and risk scores to construct a nomogram to predict the prognosis of HCC patients (Fig. 3C). The ROC curve confirmed the good predictive efficacy of nomogram for the overall survival time of HCC patients at 1, 3, and 5 years, with AUC values of 0.637, 0.601, and 0.609 at 1, 3, and 5 years, respectively(Fig. 3B). Calibration curves confirm that the nomogram has good predictive performance for overall survival time at 1, 3, and 5 years in HCC patients (Fig. 3D). Decision curves confirmed that the nomogram constructed from stage and risk scores was superior to the nomogram constructed from other factors (Fig. 3E).

**Table 2:** Results of univariate and multivariate analyses of clinical characteristics and risk scores in the TCGA LIHC dataset

Characteristics	Univariate analysis		Multivariate analysis	
	Hazard ratio (95% CI)	*P* value	Hazard ratio (95% CI)	*P* value
Age	1.011 (0.997–1.026)	0.125		
Gender				
Female	Reference			
Male	0.792 (0.545–1.150)	0.221		
G stage				
G1 + G2	Reference			
G3 + G4	1.186 (0.819–1.716)	0.366		
M stage				
M0	Reference			
MX	1.392 (0.915–2.117)	0.122		
N stage				
N0	Reference			
NX	1.228 (0.845–1.961)	0.239		
Stage				
StageI + Stage II	Reference			
Stage III + Stage IV	2.409 (1.664–3.487)	< 0.001	2.177 (1.489–3.181)	< 0.001
RiskScore	1.081 (1.059–1.103)	< 0.001	1.071 (1.049–1.1095)	< 0.001

### Comparison of prognostic signature and external prognostic signature

To further verify the superiority of the prognostic signature, we compare the ROC curve of our prognostic signature with the ROC curve of the prognostic signature constructed by Shi et al. (Shi et al. 2022) based on EMT-related genes. It is found that not only the AUC of our prognostic signature in the TCGA training set (AUC of 1 year: 0.785, AUC of 3 years: 0.698) and ICGC verification set (AUC of 1 year: 0.754, AUC of 3 years: 0.676) are better than those of the prognostic signature constructed by Shi et al. (TCGA: AUC of 1 year: 0.767, AUC of 3 years: 0.680; ICGC: AUC of 1 year: 0.677, AUC of 3 years: 0.689). Subsequently, we compared the ROC curve of our prognostic signature with that of the prognostic signature constructed by Xu et al. (Xu et al. 2021b) based on EMT-related genes and found that our prognostic signature was superior to their prognostic signature in predicting 1-year survival of HCC patients (1-year AUC: 0.76). It is further confirmed that our prognostic signature based on 6 EMT-related genes has stronger advantages.

### Results of functional enrichment analysis of DEGs

**Fig. 4:**
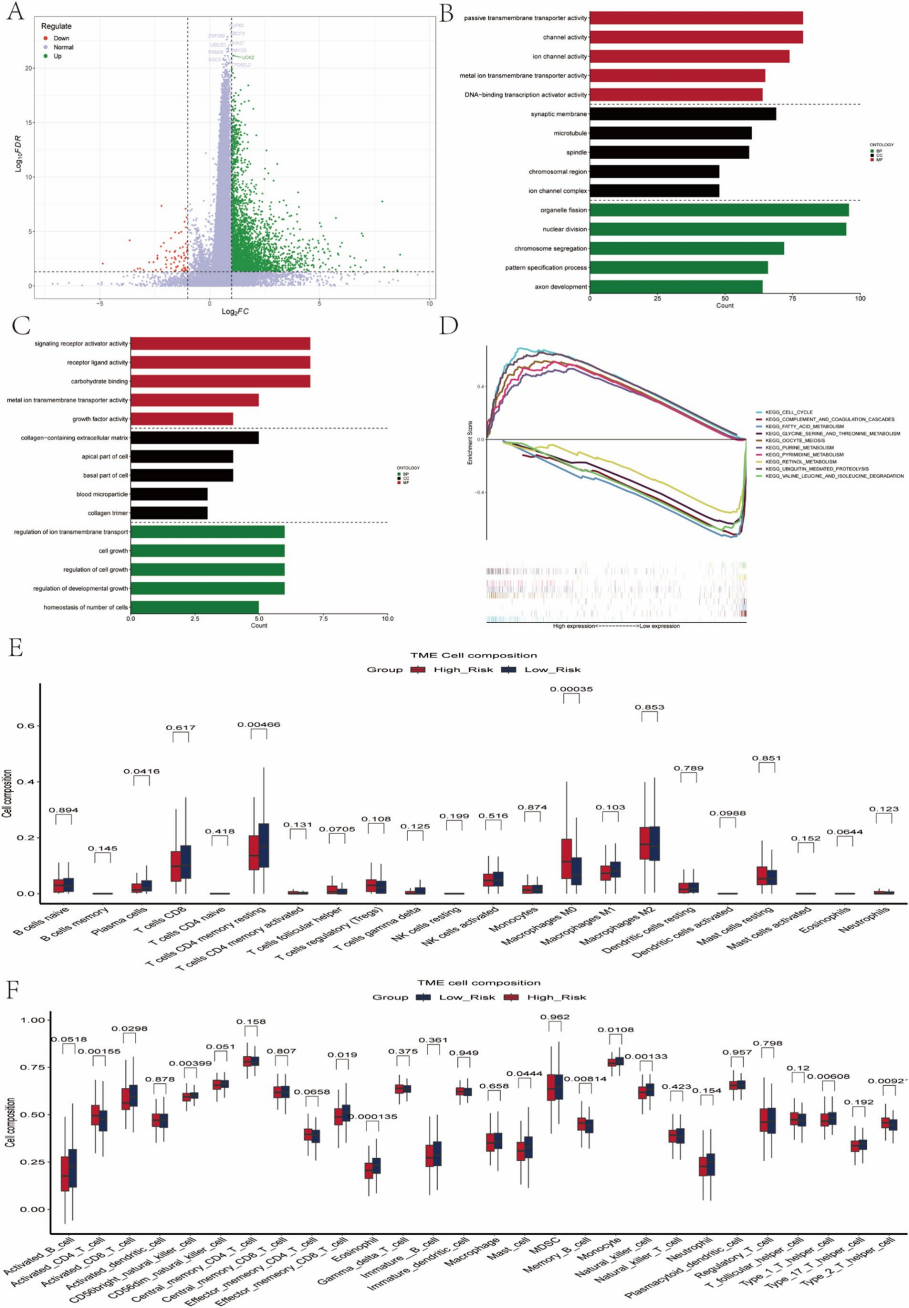
Differences between molecular characteristics between high and low risk groups (**A**) Volcano map showing the difference genes between the two groups. (**B**) Results of GO functional enrichment analysis of genes highly expressed in the high-risk group. (**C**) Results of GO functional enrichment analysis of genes highly expressed in the low-risk group. (**D**) Differences in GSEA enrichment analysis results between the high-risk and low-risk groups. (**E**) The fractions of different immune cells between high- and low-risk groups. (**F**) ssGSEA analysis shows differences in the proportions of 28 immune cell species between high and low risk groups

To explore the underlying mechanisms behind the superior predictive performance of the prognostic signature, we first performed differential analysis between the high-risk and low-risk groups in the TCGA dataset and screened for differentially expressed genes by setting the criteria of log2FC <  − 1 or log2FC > 1 and adjust. *p*-value < 0.05. By the screening criteria, there were 3175 highly expressed genes in the high-risk group, while there were 72 highly expressed genes in the low-risk group (Fig. 4A). We performed GO functional enrichment analysis on the genes that were highly expressed in the high-risk group and those that were highly expressed in the low-risk group to investigate the molecular mechanisms involved in these differential genes. The results of GO functional enrichment analysis showed that the genes highly expressed in the high-risk group were mainly enriched in passive transmembrane transporter activity, channel activity, and ion channel activity (Fig. 4B), while the genes highly expressed in the low-risk group were mainly enriched in signaling receptor activator activity and receptor-ligand activity (Fig. 4C). We used the GSEA software to analyze the bulk RNA sequencing data from 365 HCC patients. Subsequently, we further compared the differences between the high- and low-risk groups in terms of functional enrichment. In the high-risk group, GSEA KEGG enrichment analysis showed that the high-risk group was significantly enriched in OOCYTE_MEIOSIS, UBIQUITIN_MEDIATED_PROTEOLYSIS, CELL_CYCLE, SPLICEOSOME, RNA_DEGRADATION. While the low-risk group was enriched in COMPLEMENT_AND_COAGULATION_CASCADES, GLYCINE_SERINE_AND_THREONINE_METABOLISM, PRIMARY_BILE_ACID_BIOSYNTHESIS, FATTY_ACID_METABOLISM, RETINOL_METABOLISM (Fig. 4D)**.**

### Relationship between prognostic signature and immune cells infiltration

As immune cells are closely related to the immune microenvironment, we investigated the relationship between risk scores and immune cells by analyzing the RNA sequencing data. We calculated the percentage of immune cell infiltration in the tumor microenvironment of 365 HCC patients using the CIBERSORT function of the R software. We found a higher proportion of macrophages and a lower proportion of plasma cells and T cells CD4 memory resting in the high-risk group compared to the low-risk group (Fig. 4E). In addition, we investigated the relationship between risk scores and 28 immune cell types using ssGSEA enrichment scores. The results revealed that activated CD4_T_ cells and type 2_T_helper_celmultiple functional T cells were significantly enriched in the high-risk group, while activated CD8_T_ cells and CD56bright_natural_killer_ cells were more predominant in the low-risk group (Fig. 4F). Using Kaplan–Meier, we compared the survival differences between the highly infiltrated M0 macrophages group and the low-infiltrated group, and the results showed that the survival time of the highly infiltrated M0 macrophages group was significantly worse than that of the low-infiltrated group (*p* < 0.05) (Figure S6A). Similarly, we compared the survival difference between the high-infiltration CD8 T cells and the low-infiltration group. The results showed that the overall survival time of the high-infiltration group was significantly longer than that of the low-infiltration group (*p* < 0.05) (Figure S6B). We then calculated the relationship between StromalScore, ImmuneScore, ESTIMATEScore, TumorPurity, and the prognostic signature, and the results showed that ESTIMATEScore and StromalScore were higher in the low-risk group, while TumorPurity was higher in the high-risk group (Figure S6C-S6F).

### Relationship between TMB and MSI and prognostic signature

**Fig. 5:**
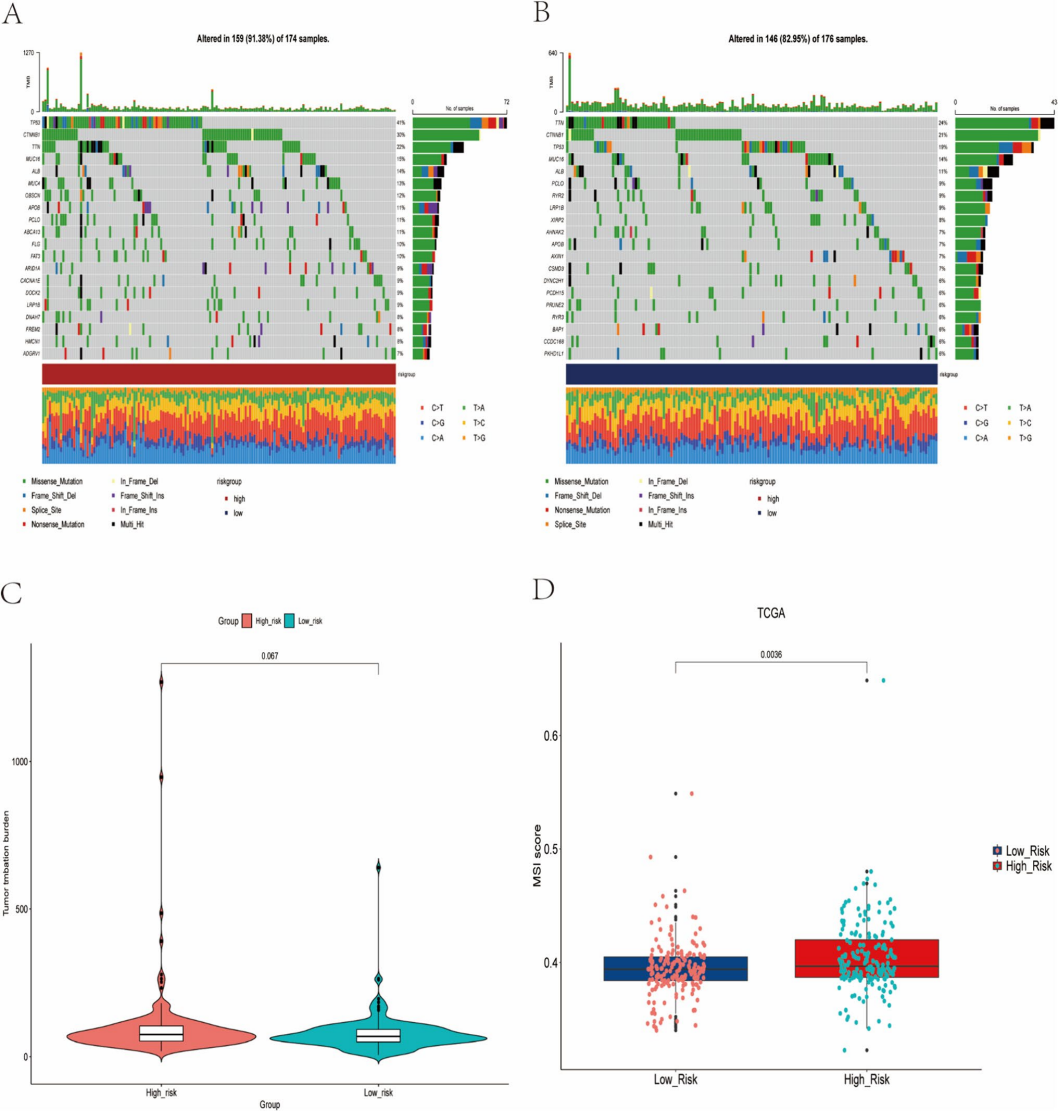
Gene mutation landscape of tumor cells in high-risk and low-risk groups. (**A**) The waterfall diagram shows the gene mutation of tumor cells in the high-risk group. (**B**) The waterfall diagram shows the gene mutation of tumor cells in the low-risk group. (**C**) Difference of tumor mutation burden between high-risk group and low-risk group. (**D**) Differences in microsatellite instability between high-risk and low-risk groups

We used mutation data to examine the relationship between the prognostic signature and mutations. The high-risk group had a higher mutation frequency(91.38%) than the low-risk group(82.95%). The top ten mutational genes in the high-risk group were TP53, CTNNB1, TTN, MUC16, ALB, MUC4, OBSCN, APOB, PCLO, and ABCA13 (Fig. 5A). The top ten mutated genes in the low-risk group were TTN, CTNNB1, TP53, MUC16, ALB, PCLO, and RYR (Fig. 5B). To investigate the relationship between prognostic signature and TMB, we compared the difference between the high-risk and low-risk groups in terms of TMB, and curiously, there was no difference in TMB between the two groups (*p* > 0.05). However, we found that the median TMB values were higher in the high-risk group than in the low-risk group (Fig. 5C), suggesting that the high-risk group may be more responsive to immunotherapy. We further explored the relationship between MSI and prognostic signature, and interestingly, we found that MSI was significantly higher in the high-risk group than in the low-risk group (Fig. 5D), further suggesting that the high-risk group may be more responsive to immunotherapy and more suitable for immunotherapy.

### Prediction of immunotherapy response

**Fig. 6:**
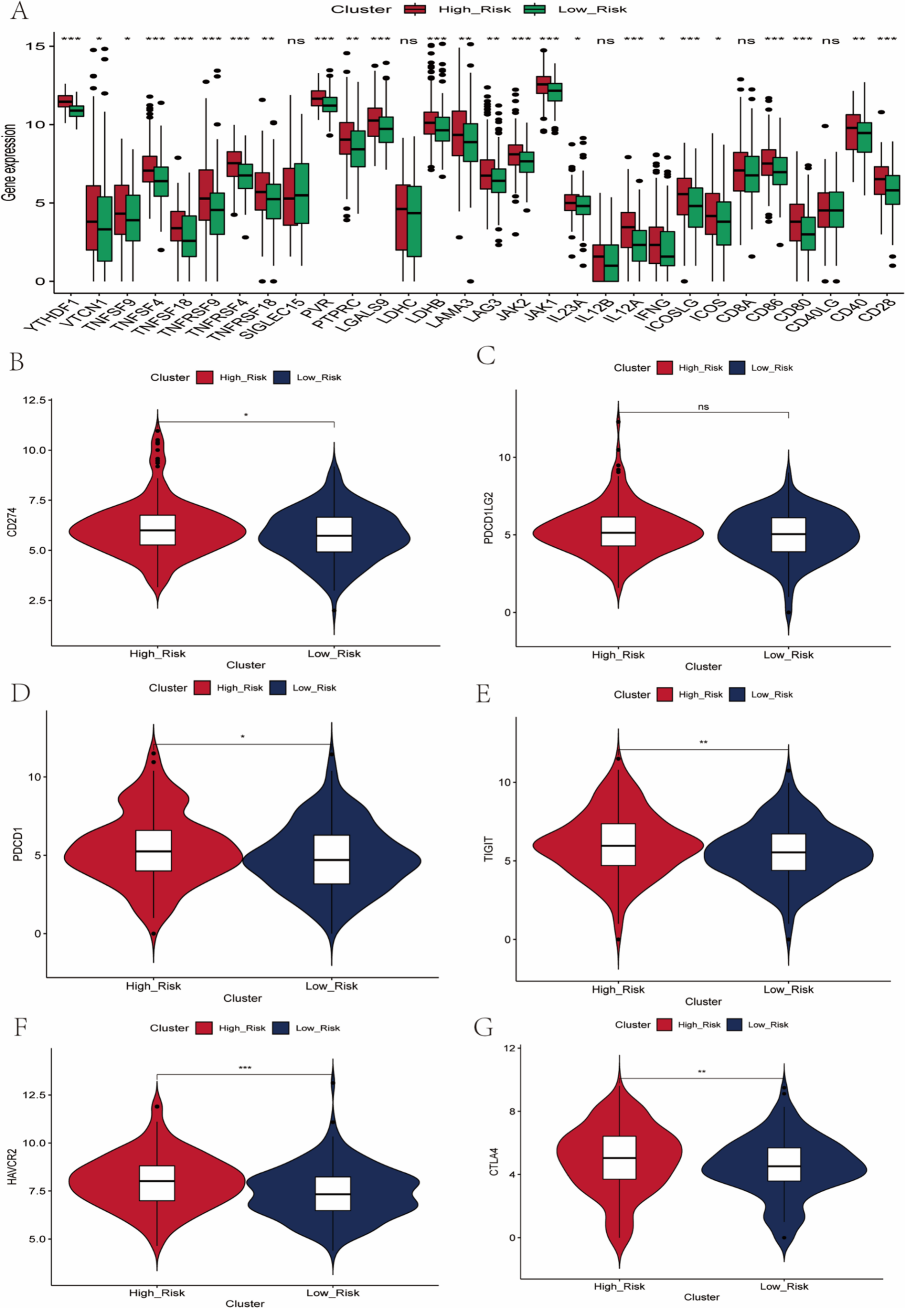
Differences in immunosuppression in the tumor microenvironment between high- and low-risk groups (**A**) Differences in 30 immune checkpoint-associated genes between high- and low-risk groups in the TCGA LIHC dataset. (**B**-**G**) Differences in six immune checkpoint-related genes (PD-L1, PD-L2, PD1, TIM-3, TIGIT, CTLA4) commonly used in immunotherapy for HCC between high- and low-risk groups

**Fig. 7:**
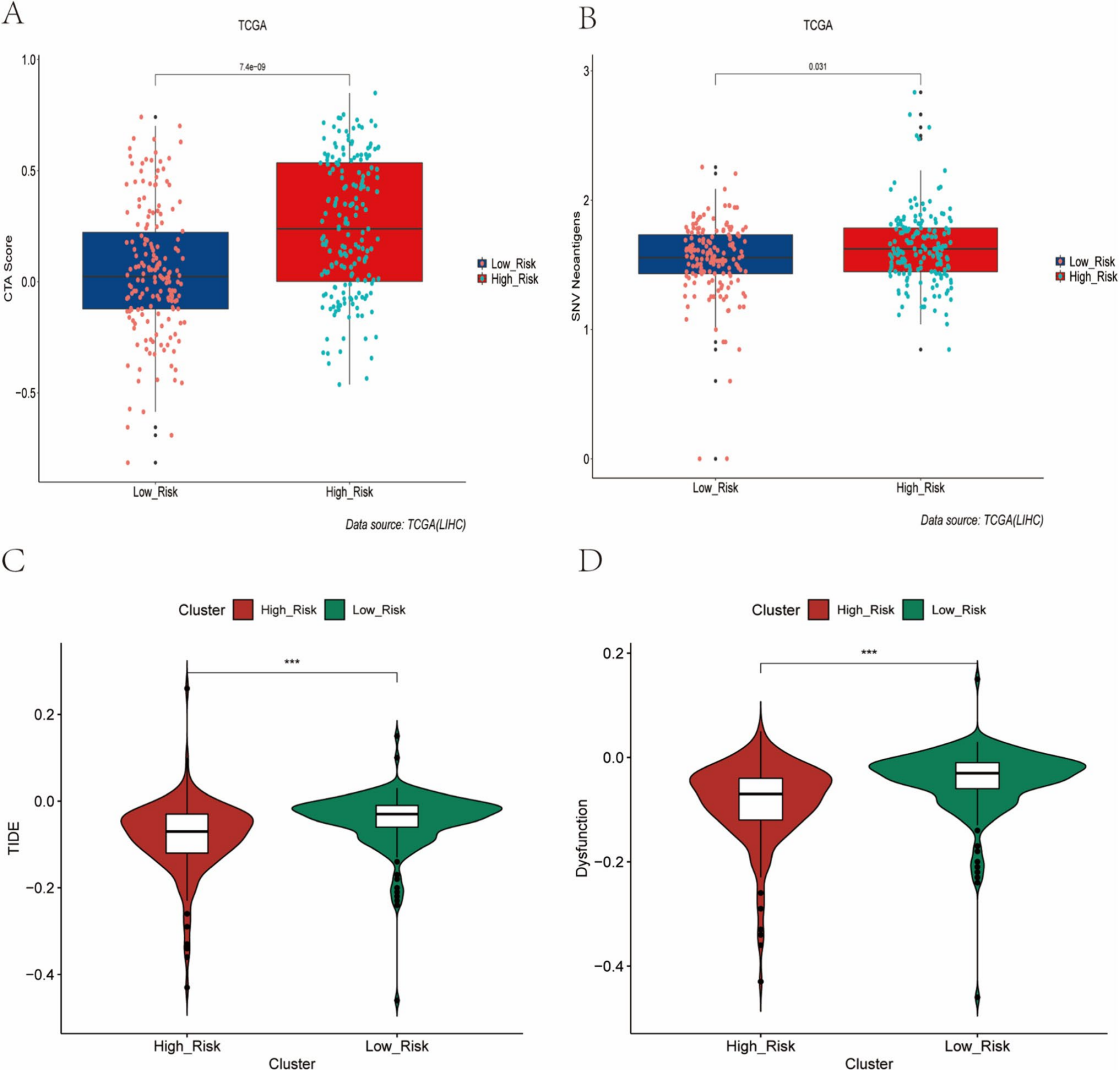
Differences in immunotherapy response assessment metrics between high- and low-risk groups (**A**) Box plot showing the difference in CTA Score between the high risk and low risk groups. (**B**) Box plot showing the difference in SNV Neoantigens Score between the high risk and low risk groups. (**C**) Differences in Tumor Immune Dysfunction and Exclusion Between Patients in High and Low Risk Groups in the TCGA LIHC Dataset (**D**) Differences in Dysfunction scores between patients in the high- and low-risk groups in the TCGA LIHC dataset

Immune checkpoint gene expression is important in predicting immunotherapy response in patients with HCC. We compared the differences in mRNA expression of immune checkpoint–associated genes between the high-risk and low-risk groups and the results showed that the mRNA expression level of most immune checkpoint–related genes was higher in the high-risk population (Fig. 6A). Subsequently, we compared the mRNA expression levels of immune checkpoint–related genes commonly used in HCC treatment, including PD-L1, PDCD1LG2, PDCD1, TIGIT, TIM-3, and CTLA4, which we found to be overexpressed in the high-risk group, implying that the high-risk group may respond to immunotherapy (Fig. 6B–G). We compared the differences between the high-risk and low-risk groups in terms of CTA scores and SNV scores, and the results showed that the CTA scores and SNV scores were significantly higher in the high-risk group than in the low-risk group (Fig. 7A–B). A growing number of studies have confirmed that TIDE scores and T cell dysfunction scores can be used to assess immunotherapy response, and higher tumor TIDE prediction scores are associated not only with poorer efficacy of immune checkpoint suppression therapy, but also with poorer patient survival under anti-PD1 and anti-CTLA4 therapy. Interestingly, we found lower TIDE scores and T cell dysfunction scores in the high-risk group compared to the low-risk group (Fig. 7C–D), while suggesting that patients in the high-risk group have fewer immune cells in a non-functional state in the tumor microenvironment and are more likely to be more responsive to immunotherapy. Patients in the high-risk group in the prognostic signature had significantly higher levels of PD-L1 mRNA expression; TMB, MSI, and SNV neoantigen; and lower levels of TIDE scores and T cell dysfunction scores, suggesting that tumors in the high-risk group are highly immunogenic and can activate immune cells to recognize tumors, but high PD-L1 expression induces suppression of anti-tumor immunity in high-risk group patients. We used external independent immunotherapy data to validate our findings, which showed a higher percentage of patients with CR/PR after immunotherapy in the high-risk group (Figure S7), further confirming that patients in the high-risk group are more suitable for immunotherapy. Overall, these results further suggest a correlation between the prognostic signature constructed from EMT-related genes and immunotherapy. Patients in the high-risk group identified based on the prognostic signature had a significant immune suppressed state of immune infiltrating lymphocytes in the tumor microenvironment, and immunotherapy may be more appropriate and responsive.

### qRT-PCR confirmed overexpression of six EMT-related genes in HCC tissues

**Fig. 8:**
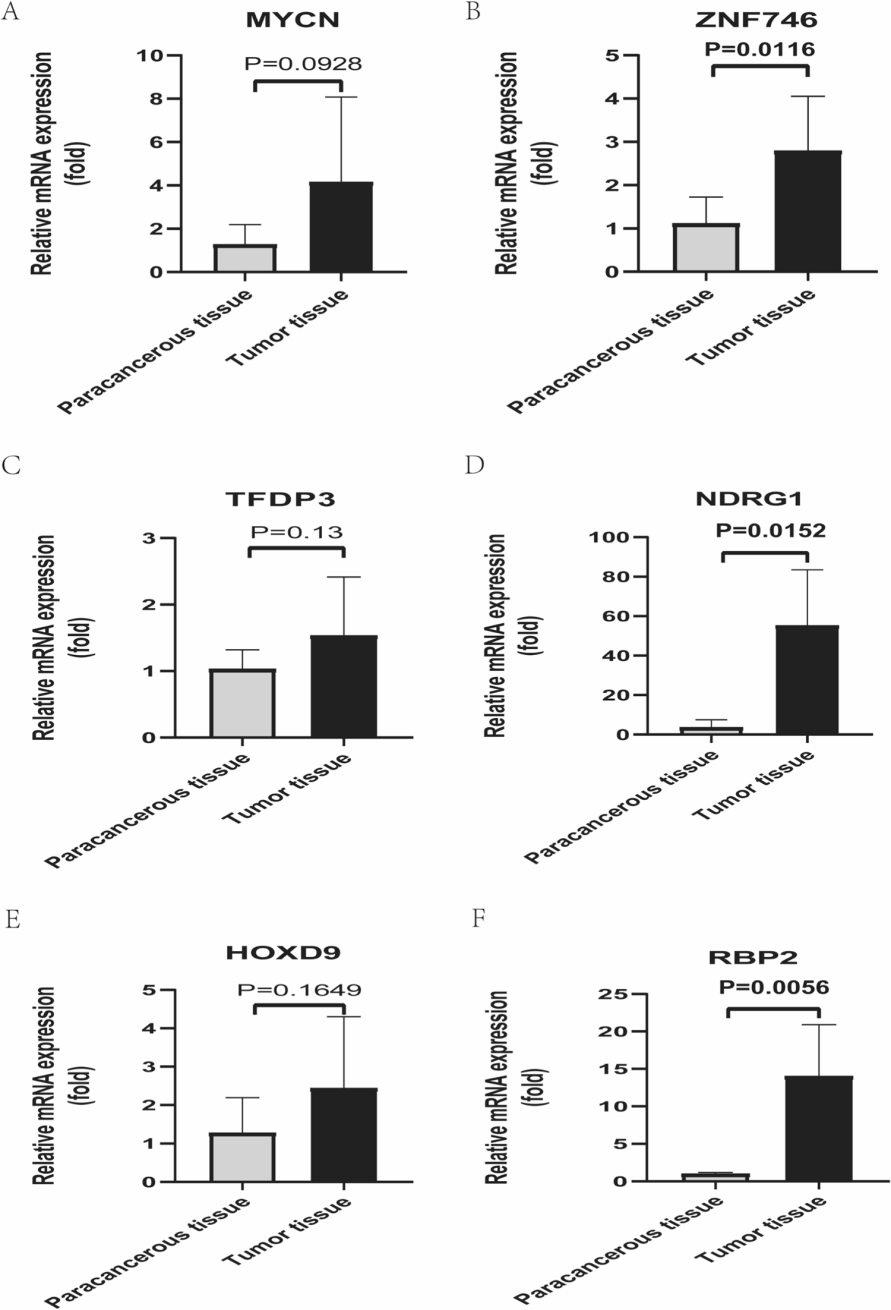
The mRNA expression of six EMT related genes was confirmed by qRT-PCR in HCC tissues. (**A**-**F**) The results of qRT-PCR confirmed that the mRNA expression level of the six EMT related genes in the prognostic signature(MYCN, ZNF746, TFDP3, NDRG1, HOXD9, and RBP2) were overexpressed compared with the adjacent tissues

To further validate the prognostic signature, we analyzed the tissue samples of 6 HCC patients. The results of qRT-PCR confirmed that the mRNA expression level of the six EMT-related genes in the prognostic signature(MYCN, ZNF746, TFDP3, NDRG1, HOXD9, and RBP2) were overexpressed compared with the adjacent tissues (Fig. 8). These results further confirmed the robustness of the prognostic signature.

## Discussion

HCC is the sixth most common cancer in the world, and it is the fourth most lethal (Hwang et al. 2021). The introduction of immune checkpoint inhibitors in recent years has dramatically changed the treatment of hematologic and solid tumors, including HCC (Ingles et al. 2019; Finkelmeier et al. 2018). However, significant problems such as low immune response rates remain in clinical practice, so it is critical to screen potentially immune-responsive patients for HCC treatment by using biomarkers. A growing number of studies have confirmed the link between EMT and tumor progression and metastasis (Tao et al. 2020). However, the association between EMT and immune cell infiltration in the tumor microenvironment, somatic mutations, expression of immune checkpoint–related genes, and the immunotherapeutic response has not been adequately demonstrated.

In the present study, we constructed a novel prognostic model based on EMT-related associated genes through a comprehensive analysis of data from TCGA and GEO databases. To explore the molecular mechanisms behind the prognostic model, we performed a comprehensive analysis of the expression levels of immune cell infiltration, somatic mutations, TMB, MSI, TIDE, and immune checkpoint–related genes. The results show that the prognostic model can effectively predict not only the prognosis of HCC patients but also the response to immunotherapy and screen the population suitable for immunotherapy. The validity and robustness of the prognostic model were validated in multiple external independent datasets.

The EMT-related genes that make up our prognostic signature have all been identified in a variety of tumors. MYCN, for example, has been shown to be a prognostic factor for HCC as well as a therapeutic target for acyclic retinoid (Qin et al. 2018a). ZNF746 has been shown to promote HCC progression and to be a target for HCC-targeted therapy (Kim et al. 2021). In HCC, TFDP3 can inhibit apoptosis induced by hypoxia-inducible factor 2 (HIF-2) and promote tumorigenesis (Sun et al. 2013). Cheng et al. demonstrated that NDRG1 plays an important role in HCC metastasis and can be used as a novel molecular marker to predict HCC recurrence (Cheng et al. 2011). HOXD9 has been shown to promote HCC epithelial-mesenchymal transformation and tumor cell metastasis (Lv et al. 2015). Wang et al. demonstrated that RBP2 expression is an independent poor prognostic factor for DFS and OS in HCC, that it is closely related to tumor angiogenesis, and that RBP2 is a new potential therapeutic target for HCC (Wang et al. 2017). In this study, we collected 365 clinical samples from HCC patients, including 56 HCC tumor tissues and 4662 paracancerous tissue samples. We verified that 6 EMT genes were overexpressed in tumor tissues using qRT-PCR.

In this study, the prognostic model proved to be a useful tool in predicting the prognosis of HCC patients in the training set and validation set. The superior predictive performance of the prognostic model prompted us to further explore the molecular mechanisms behind it. We first performed GO and KEGG functional enrichment analysis, which showed that the genes highly expressed in the high-risk group were mainly enriched in passive transmembrane transporter activity, channel activity, and ion channel activity signaling pathways. The results of KEGG functional enrichment analysis showed that the genes highly expressed in the high-risk group were mainly enriched in MEIOSIS, UBIQUITIN_MEDIATED_PROTEOLYSIS, and CELL_CYCLE signaling pathways, which play an important role in tumor appreciation and invasion. These signaling pathways partially explain the poorer prognosis of patients in the high-risk group. The interactions between tumor cells, stromal cells, and multiple immune cells in the tumor microenvironment promote tumor immune escape, deterioration, increased invasiveness, and treatment resistance, and therefore, it is essential to elucidate the mechanisms by which various aspects of the TME network coordinate or inhibit immune responses to reverse immune drug resistance. In this study, we comprehensively profiled the tumor microenvironment in the high-risk and low-risk groups, and we found a higher proportion of activated CD4_T_cell, and macrophages, and a lower proportion of activated CD8_T_cells and CD56bright_natural_killer_cells compared to the low-risk group. In this study, we comprehensively profiled the tumor microenvironment in the high-risk and low-risk groups, and we found a higher proportion of activated CD4_T_cell, and macrophages, and a lower proportion of activated CD8_T_cells and CD56bright_natural_killer_cells compared to the low-risk group. Cells with immunotoxic function were highly infiltrated in the low-risk group, which partly explains the better prognosis of the low-risk group.

Tumor mutational burden (TMB) quantifies the presence of nonsynonymous single nucleotide variants in tumor cells that produce different proteins that are recognized as neo-antigens by the immune system and boost the anticancer immune response (Yarchoan et al. 2017; Qin et al. 2018b; Teng et al. 2018). Several studies have found that having a high TMB is associated with a higher rate of immunotherapy response in melanoma and NSCLC (Hellmann et al. 2018; Goodman et al. 2017). In our study, the median TMB was higher in the high-risk group than in the low-risk group, indicating that the high-risk group had a higher tumor mutation load and mutation frequency. MSI refers to the phenomenon of altered microsatellite sequence length due to insertion or deletion mutations during DNA replication, manifested by growth or truncation of microsatellite sequences in some tumor cells, often caused by defective mismatch repair (MMR) function. MSI has been demonstrated in colorectal cancer as an effective biomarker for immune checkpoint inhibitors. We compared the differences between the high-risk and low-risk groups in MSI scores and showed that the MSI scores were significantly higher in the high-risk group than in the low-risk group (*p* < 0.05). Therefore, combining these results, we inferred that patients with HCC in the high-risk group had higher TMB and MSI and might be more responsive to immunotherapy and more suitable for immunotherapy.

The expression of immune checkpoint–related genes is the basis of immunotherapy. Interestingly, we found that the mRNA levels of most immune checkpoint–related genes showed overexpression in the high-risk group. Subsequently, we compared the mRNA expression levels of immune checkpoint–related genes commonly used in HCC treatment, including PD-L1, PDCD1LG2, PDCD1, TIGIT, TIM-3, and CTLA4, which we found to be overexpressed in the high-risk group. These results suggest that the immunosuppressive state exists in the tumor microenvironment of HCC patients in the high-risk group, and immunotherapy may reverse the immunosuppressive state and restore the killing function of immune cells.

An increasing number of studies have confirmed that CTA scores and SNV can be used as biological markers to determine the response to immunotherapy. In the present study, we compared the differences in CTA score and SNV between the high-risk and low-risk groups. Surprisingly, we found that CTA scores and SNV were significantly higher in the high-risk group than in the low-risk group. TIDE scores have been used to predict response to tumor immunotherapy, and tumor patients with high TIDE scores may not respond to immunotherapy, while patients with low TIDE scores may benefit from immunotherapy. We found that the TIDE scores in the high-risk group were significantly lower than those in the low-risk group, and these results further suggest that patients with HCC in the high-risk group are more suitable for immunotherapy.

PD-1/PD-L1 inhibitors play an important role in the treatment of certain refractory tumors by altering immune surveillance status (Yi et al. 2018). Numerous clinical studies have shown that PD-L1 expression on tumor cells or in the tumor microenvironment is positively correlated with the response rate to anti-PD-1/PD-L1 therapy (Patel and Kurzrock 2015; Grigg and Rizvi 2016), suggesting that patients with high immune checkpoint expression may benefit from immunotherapy. In the current study, patients in the high-risk group had significantly higher expression of PD-1, TIGIT, CTLA4, and TIM-3 compared to patients in the low-risk group, implying that the high-risk group in the prognostic signature may represent the immunogenic microenvironment, and thus, we inferred that HCC patients with high-risk scores might respond better to PD-1, TIGIT, CTLA4, and TIM-3 antibodies. As a result, PD-1, TIGIT, CTLA4, and TIM-3 inhibitors like pembrolizumab, ipilimumab, tremelimumab, and sabatolimab may be effective treatments for these patients.

This work has some limitations, despite the fact that our EMT prognostic signature may effectively predict survival and immunotherapy response in patients with HCC. Due to the fact that our signature was constructed utilizing public TCGA, GEO, and ICGC databases, additional cancer immunology, immunotherapy, and basic investigations are required to evaluate the predictive potential of the characteristics and discover the biological functions of EMT-related genes.

## Conclusions

Our novel prognostic signature, constructed based on EMT-related genes, can effectively predict prognosis and immunotherapy response in HCC patients. This predictive efficacy has been demonstrated in multiple external independent datasets. In the future, as our research progresses, our prognostic signature will be validated in clinical samples and will become a useful tool for clinicians to screen for people suitable for immunotherapy.

## Supplementary Information

Below is the link to the electronic supplementary material.

Supplementary file1 (DOCX 4721 KB)

Supplementary file2 (DOCX 17 KB)

## Data Availability

Gene expression profiles, clinical information, and mutation data of HCC in this study are available from the public database (TCGA, https://portal.gdc.cancer.gov/;ICGC,https://dcc.icgc. org/). The EMT-related gene set in this study are available from the Epithelial-Mesenchymal Transition Gene Database (http://dbemt.bioinfo-minzhao.org/) website.

## References

[CR1] Akinyemiju T, Abera S, Ahmed M (2017). The burden of primary liver cancer and underlying etiologies from 1990 to 2015 at the global, regional, and national level: results from the global burden of disease study 2015. JAMA Oncol.

[CR2] Cheng J, Xie HY, Xu X (2011). NDRG1 as a biomarker for metastasis, recurrence and of poor prognosis in hepatocellular carcinoma. Cancer Lett.

[CR3] Chidambaranathan-Reghupaty S, Fisher PB, Sarkar D (2021). Hepatocellular carcinoma (HCC): epidemiology, etiology and molecular classification. Adv Cancer Res.

[CR4] Colaprico A, Silva TC, Olsen C (2016). TCGAbiolinks: an R/bioconductor package for integrative analysis of TCGA data. Nucleic Acids Res.

[CR5] EASL-EORTC clinical practice guidelines (2012). management of hepatocellular carcinoma. J Hepatol.

[CR6] Finkelmeier F, Waidmann O, Trojan J (2018). Nivolumab for the treatment of hepatocellular carcinoma. Expert Rev Anticancer Ther.

[CR7] Goodman AM, Kato S, Bazhenova L (2017). Tumor mutational burden as an independent predictor of response to immunotherapy in diverse cancers. Mol Cancer Ther.

[CR8] Grigg C, Rizvi NA (2016). PD-L1 biomarker testing for non-small cell lung cancer: truth or fiction?. J Immunother Cancer.

[CR9] Hänzelmann S, Castelo R, Guinney J (2013). GSVA: gene set variation analysis for microarray and RNA-seq data. BMC Bioinforma.

[CR10] Hellmann MD, Ciuleanu TE, Pluzanski A (2018). Nivolumab plus ipilimumab in lung cancer with a high tumor mutational burden. N Engl J Med.

[CR11] Hwang YJ, Lee Y, Park H (2021). Prognostic significance of viable tumor size measurement in hepatocellular carcinomas after preoperative locoregional treatment. J Pathol Transl Med.

[CR12] Ingles GA, Au L, Mason R (2019). Building on the anti-PD1/PD-L1 backbone: combination immunotherapy for cancer. Expert Opin Investig Drugs.

[CR13] Johnston MP, Khakoo SI (2019). Immunotherapy for hepatocellular carcinoma: current and future. World J Gastroenterol.

[CR14] Kim H, Lee JY, Park SJ (2021). ZNF746/PARIS promotes the occurrence of hepatocellular carcinoma. Biochem Biophys Res Commun.

[CR15] Kurebayashi Y, Ojima H, Tsujikawa H (2018). Landscape of immune microenvironment in hepatocellular carcinoma and its additional impact on histological and molecular classification. Hepatol.

[CR16] Lv X, Li L, Lv L (2015). HOXD9 promotes epithelial-mesenchymal transition and cancer metastasis by ZEB1 regulation in hepatocellular carcinoma. J Exp Clin Cancer Res.

[CR17] Li X, Wang Y, Ye X (2021). Locoregional combined with systemic therapies for advanced hepatocellular carcinoma: an inevitable trend of rapid development. Front Mol Biosci.

[CR18] Newell F, Pires DSI, Johansson PA (2022). Multiomic profiling of checkpoint inhibitor-treated melanoma: identifying predictors of response and resistance, and markers of biological discordance. Cancer Cell.

[CR19] Patel SP, Kurzrock R (2015). PD-L1 expression as a predictive biomarker in cancer immunotherapy. Mol Cancer Ther.

[CR20] Pinato DJ, Guerra N, Fessas P (2020). Immune-based therapies for hepatocellular carcinoma. Oncogene.

[CR21] Pinter M, Jain RK, Duda DG (2021). The current landscape of immune checkpoint blockade in hepatocellular carcinoma: a review[J]. JAMA Oncol.

[CR22] Qin BD, Jiao XD, Zang YS (2018). Tumor mutation burden to tumor burden ratio and prediction of clinical benefit of anti-PD-1/PD-L1 immunotherapy. Med Hypotheses.

[CR23] Qin XY, Suzuki H, Honda M (2018). Prevention of hepatocellular carcinoma by targeting MYCN-positive liver cancer stem cells with acyclic retinoid. Proc Natl Acad Sci U S A.

[CR24] Rizzo A, Ricci A D, Brandi G (2021) PD-L1, TMB, MSI, and other predictors of response to immune checkpoint inhibitors in biliary tract cancer. Cancers (Basel), 13(3). 10.3390/cancers13030558. 10.3390/cancers13030558 PMC7867133 33535621

[CR25] Robinson MD, McCarthy DJ, Smyth GK (2010). edgeR: a bioconductor package for differential expression analysis of digital gene expression data. Bioinforma.

[CR26] Roudko V, Cimen BC, Greenbaum B (2021). Lynch syndrome and MSI-H cancers: from mechanisms to “off-the-shelf” cancer vaccines. Front Immunol.

[CR27] Shi Y, Wang J, Huang G (2022). A novel epithelial-mesenchymal transition gene signature for the immune status and prognosis of hepatocellular carcinoma. Hepatol Int.

[CR28] Sun HX, Xu Y, Yang XR (2013). Hypoxia inducible factor 2 alpha inhibits hepatocellular carcinoma growth through the transcription factor dimerization partner 3/ E2F transcription factor 1-dependent apoptotic pathway. Hepatol.

[CR29] Tao C, Huang K, Shi J (2020). Genomics and prognosis analysis of epithelial-mesenchymal transition in glioma. Front Oncol.

[CR30] Teng F, Meng X, Kong L (2018). Progress and challenges of predictive biomarkers of anti PD-1/PD-L1 immunotherapy: a systematic review. Cancer Lett.

[CR31] Wang ZY, Yang J, Liu CK (2017). High expression of retinoblastoma-binding protein 2 (RBP2) in patients with hepatocellular carcinoma and its prognostic significance. Med Sci Monit.

[CR32] Xu Q, Chen S, Hu Y (2021). Landscape of immune microenvironment under immune cell infiltration pattern in breast cancer. Front Immunol.

[CR33] Xu D, Wang Y, Wu J (2021). Identification and clinical validation of EMT-associated prognostic features based on hepatocellular carcinoma. Cancer Cell Int.

[CR34] Yarchoan M, Hopkins A, Jaffee EM (2017). Tumor mutational burden and response rate to PD-1 inhibition. N Engl J Med.

[CR35] Yi M, Jiao D, Xu H (2018). Biomarkers for predicting efficacy of PD-1/PD-L1 inhibitors. Mol Cancer.

[CR36] Yu G, Wang LG, Han Y (2012). clusterProfiler: an R package for comparing biological themes among gene clusters. OMICS.

[CR37] Zhang J, Dang F, Ren J (2018). Biochemical aspects of PD-L1 regulation in cancer immunotherapy. Trends Biochem Sci.

[CR38] Zhou J, Sun HC, Wang Z (2018). Guidelines for diagnosis and treatment of primary liver cancer in China (2017 Edition). Liver Cancer.

