# Supplementary material for: Construction and validation of a novel signature based on epithelial-mesenchymal transition–related genes to predict prognosis and immunotherapy response in hepatocellular carcinoma by comprehensive analysis of the tumor microenvironment
Source: Funct Integr Genomics. 2022 Dec 20;23(1):6. doi: 10.1007/s10142-022-00933-w (PMC9763151; doi:10.1007/s10142-022-00933-w)
Supplement: Supplementary file 1 — Supplementary file1 (DOCX 4721 KB) [file 10142_2022_933_MOESM1_ESM.docx]

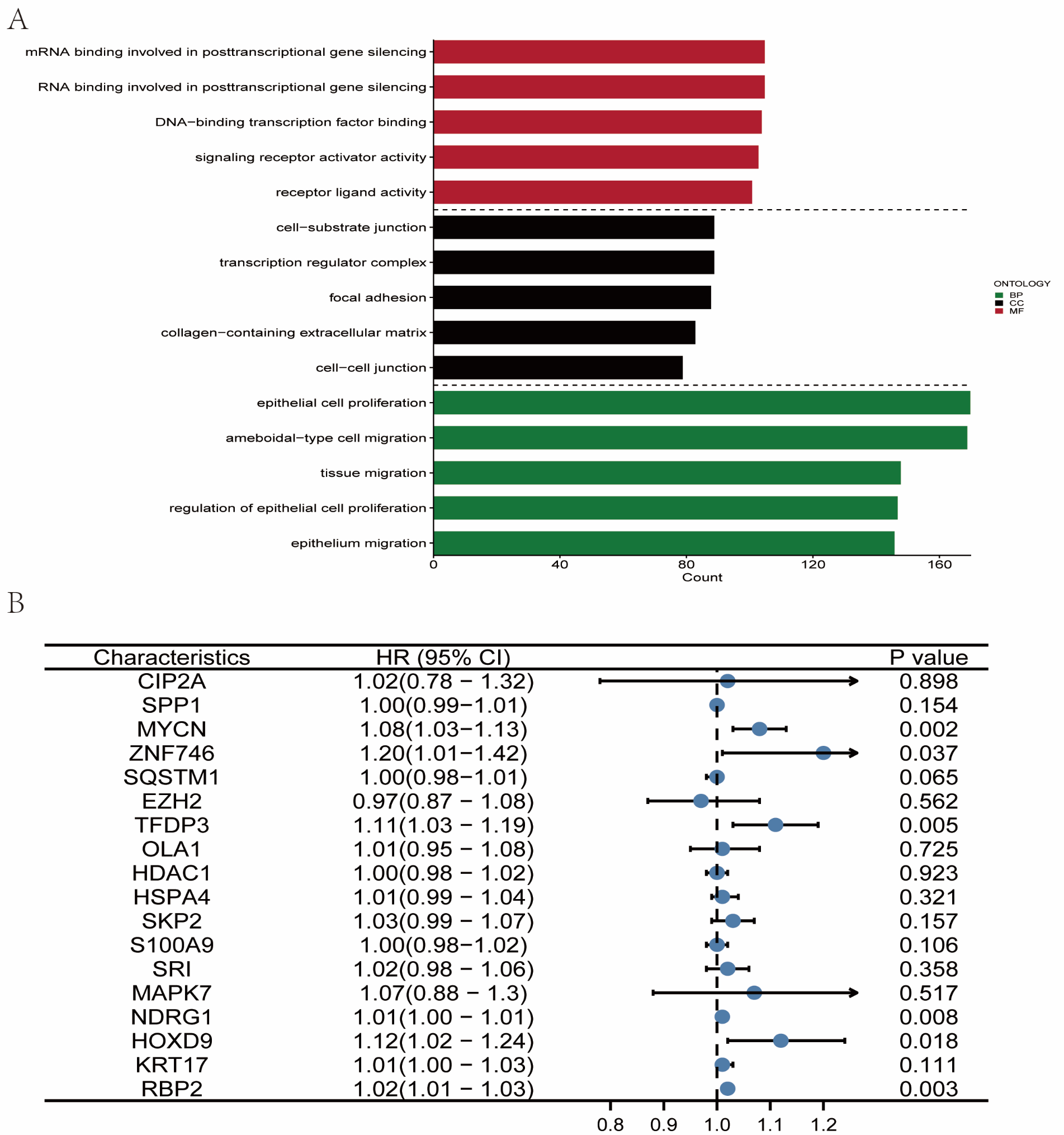


**Figure S1** GO function enrichment analysis and screening of genes for constructing prognosis signature.(A) Enrichment analysis of GO function of EMT related genes.(B) Multivariate Cox Regression Analysis Results of 16 prognostic Genes.


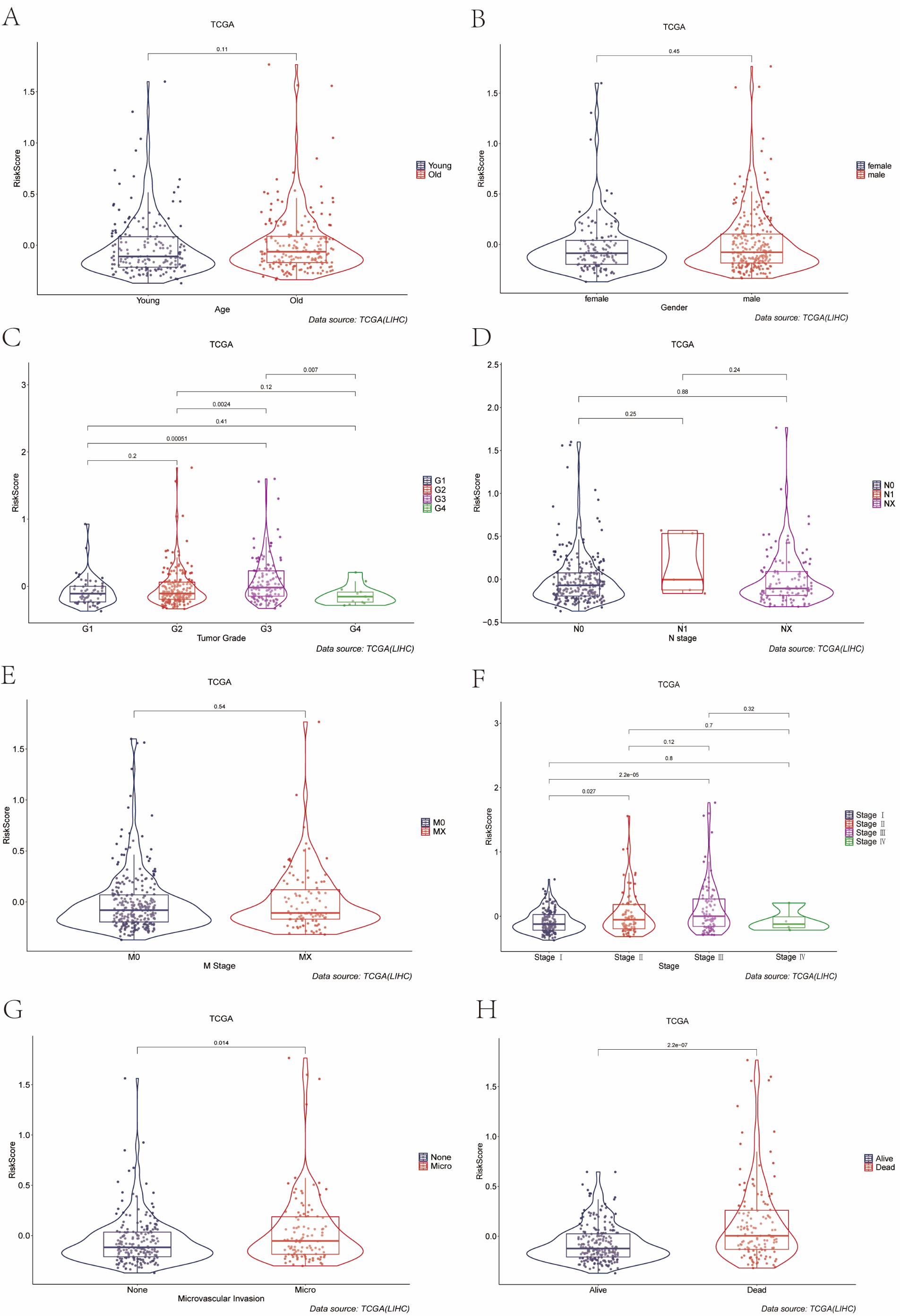


**Figure S2** Relationship between prognostic signature and clinical features in TCGA LIHC. Relationship between prognostic signature and age(A), Gender(B), Tumor Grade(C), N stage(D), M stage(E), Stage(F), Microvascular invasion(G), and Survival status(H).


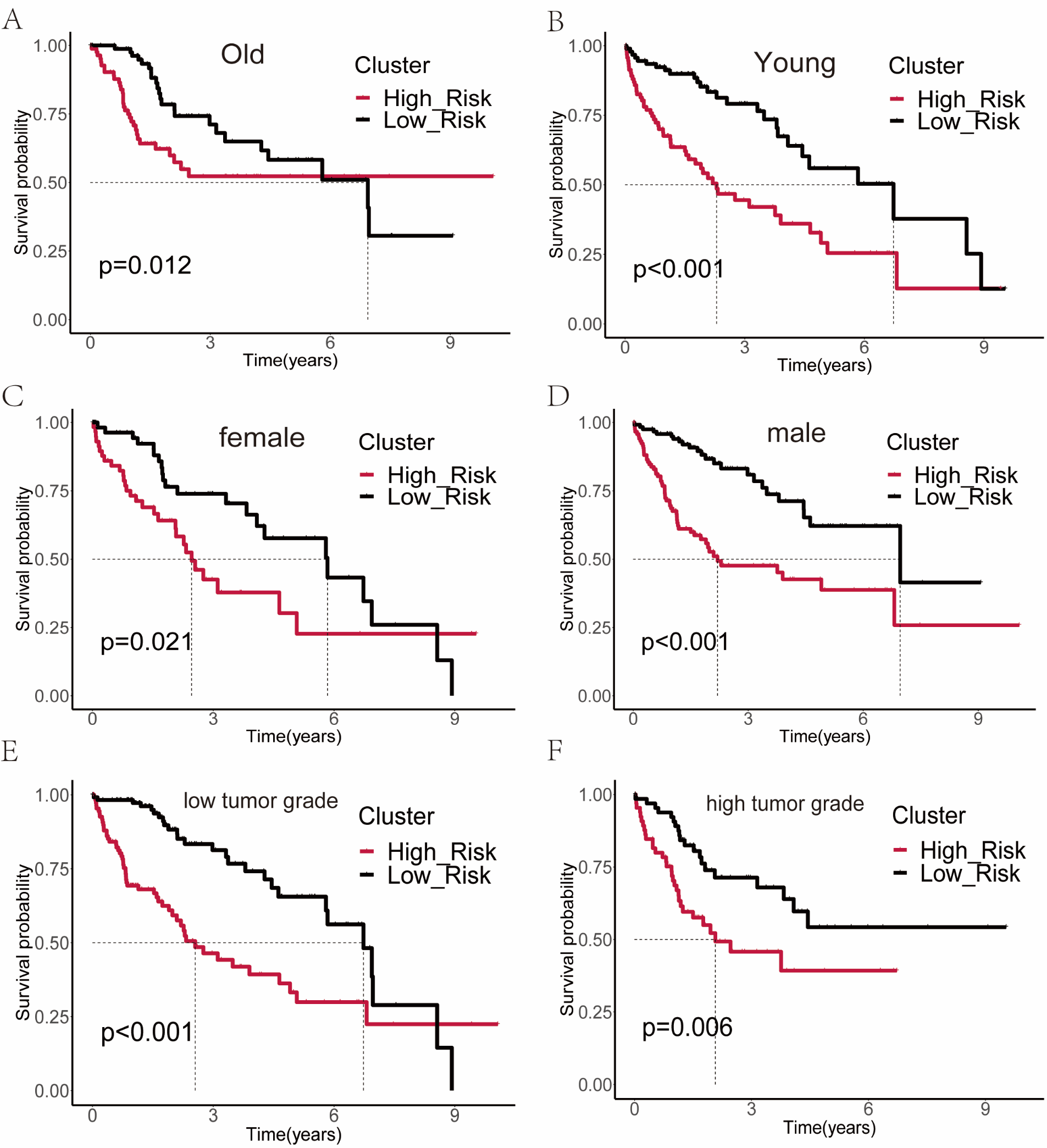


**Figure S3** Validation of the prognostic predictive ability of prognostic signature in different clinical subgroups. In different clinical subgroups, such as Old (A), Young (B), female (C), male (D), low Tumor grade (E) and high Tumor grade stage (F), the overall survival time of patients in low-risk group is significantly better than that of patients in high-risk group.


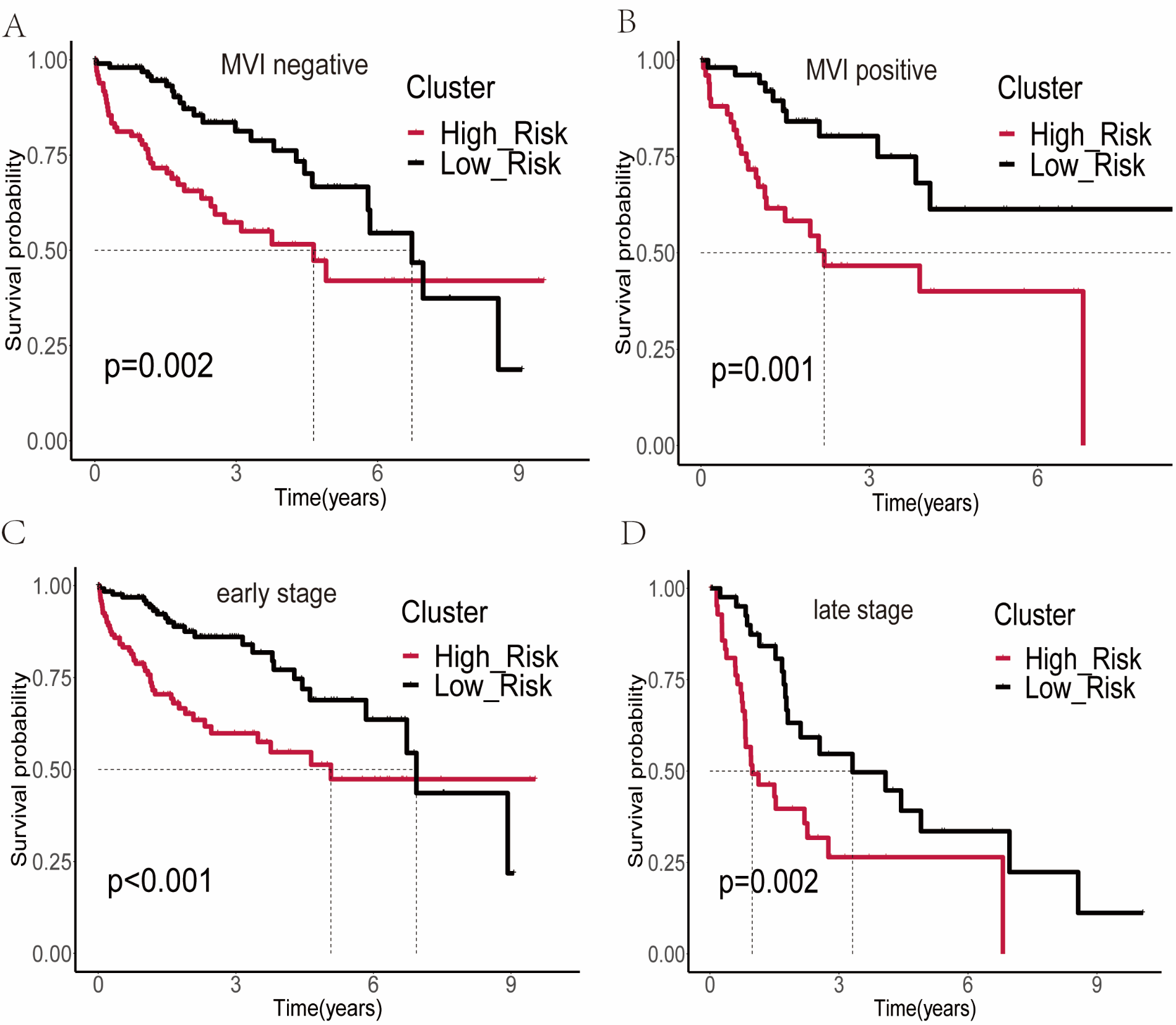


**Figure S4** The predictive efficacy of prognostic signature was verified in different clinical subgroups. Kaplan Meier curves showed that in different clinical subgroups, such as negative microvascular injection (A), positive microvascular injection (B), early stage(C) and late stage(D), the overall survival time of low-risk group was significantly better than that of high-risk group.

Ff
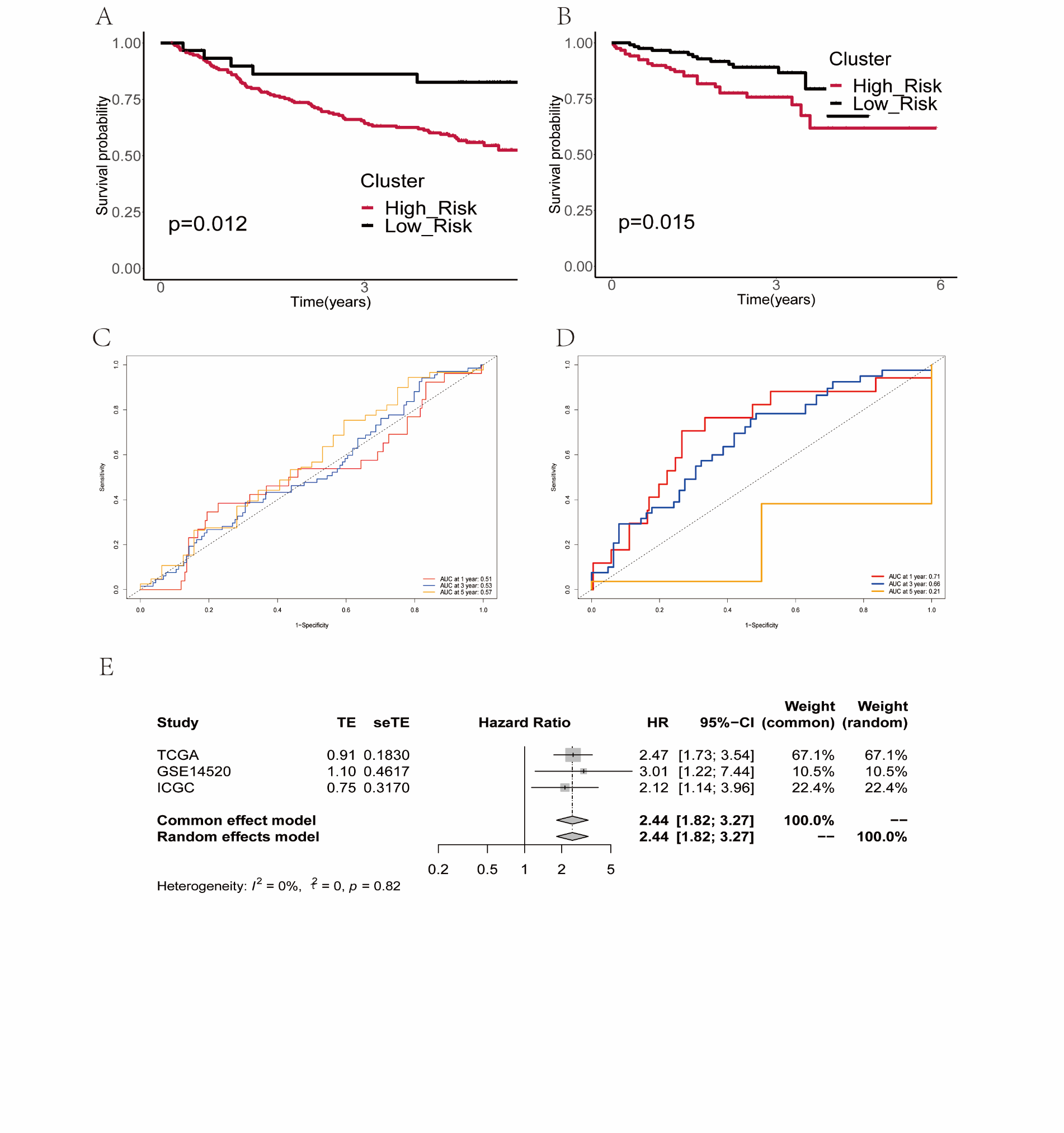


**Figure S5** Multiple external data sets were used to verify the superiority of prognostic signature. (A) Patients in the low-risk group in the GSE14250 dataset survived significantly better than those in the high-risk group. (B) Patients in the low-risk group in the ICGC data set survived significantly better than those in the high-risk group.(C) ROC curves of prognostic signature predict the risk of death at 1, 3, and 5 years in the GSE14250 dataset. (D) ROC curves of prognostic signature predict the risk of death at 1, 3, and 5 years in the ICGC dataset.(E) Meta analysis results of three data sets.


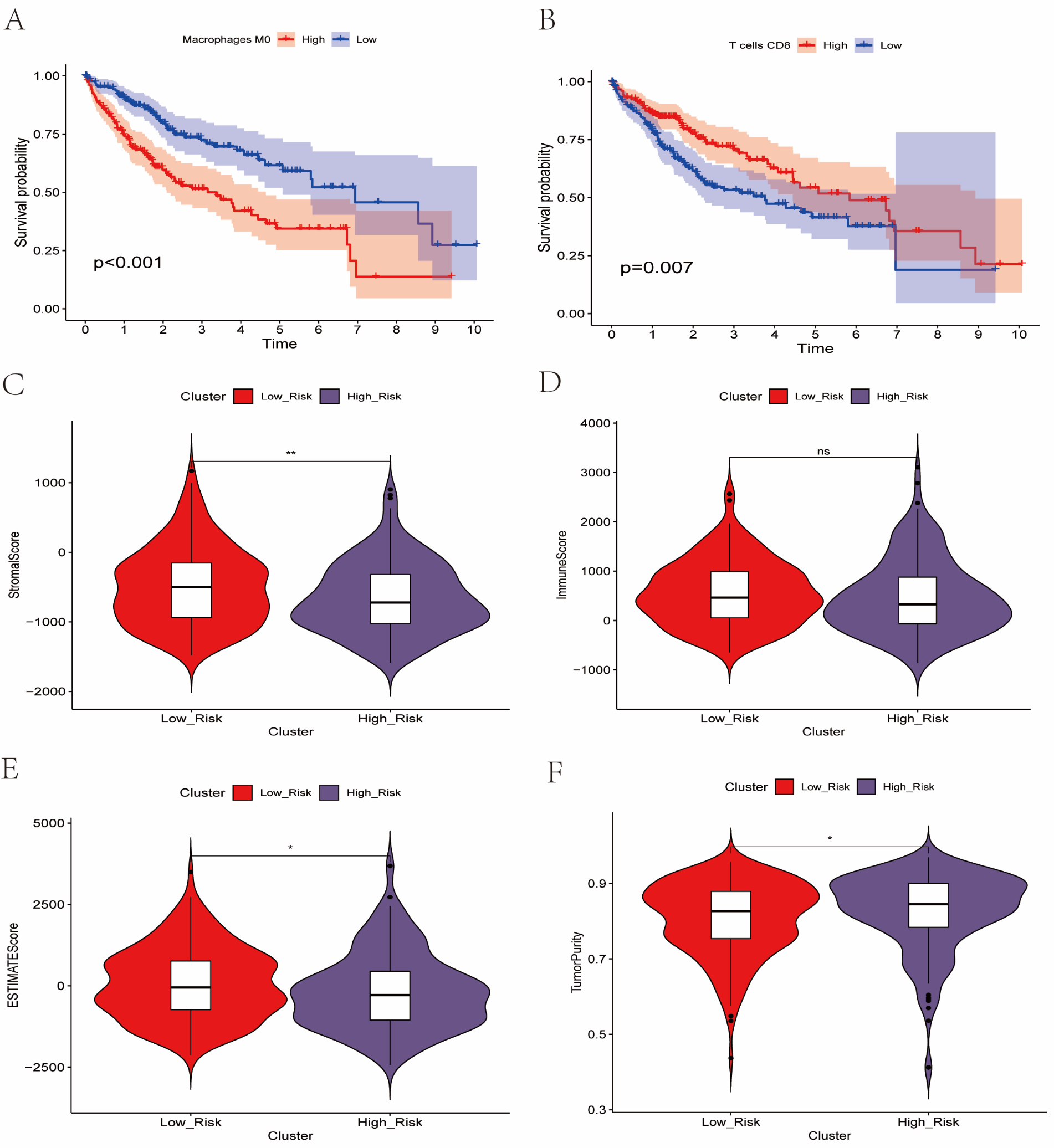


**Figure S6** Influence of proportion of immune cells infiltration on survival of patients with HCC. (A) Survival differences between the highly infiltrated M0 macrophages group and low infiltration groups.(B) Survival differences between the highly infiltrated CD8 T cell group and low infiltration groups. Differences between high-risk groups and low-risk groups in StromalScore(C), ImmunoScore(D), ESTIMATEScore(E), and TumorPurity(F).
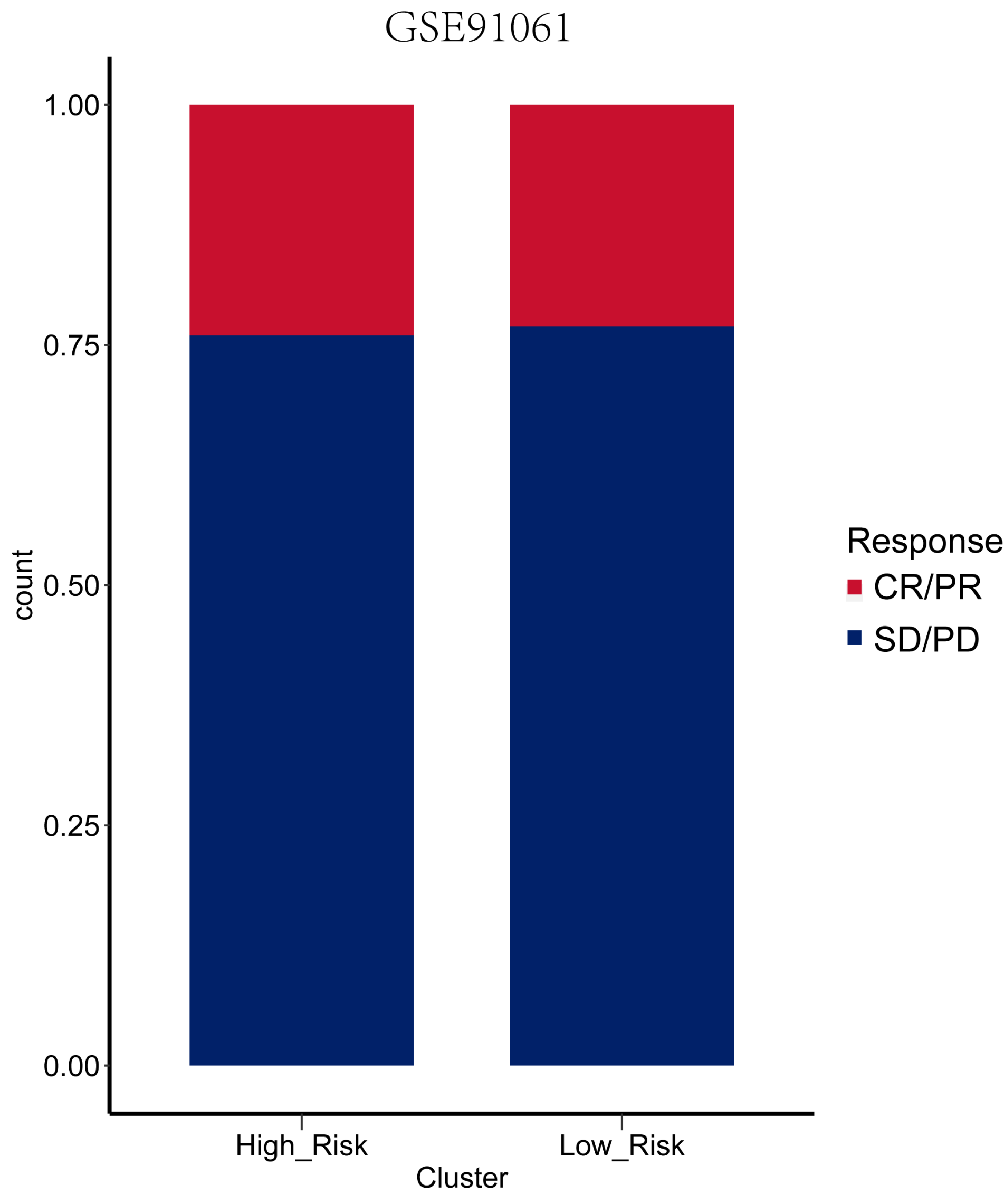


**Figure S7** External independent immunotherapy data verify that the proportion of CR/PR patients in high-risk groups is higher.
