# Supplementary material for: Construction and validation of a novel signature based on epithelial-mesenchymal transition–related genes to predict prognosis and immunotherapy response in hepatocellular carcinoma by comprehensive analysis of the tumor microenvironment
Source: Funct Integr Genomics. 2022 Dec 20;23(1):6. doi: 10.1007/s10142-022-00933-w (PMC9763151; doi:10.1007/s10142-022-00933-w)
Supplement: Supplementary file 2 — Supplementary file2 (DOCX 17 KB) [file 10142_2022_933_MOESM2_ESM.docx]

**Supplement Table 1** Clinical characteristics of HCC from multiple cohorts

| **Variables** | **GSE14520**  **(n=220)** | **ICGC**  **(n=231)** |
| --- | --- | --- |
| **Age(Year)**  **Median**  **Range** | 50  21-77 | 69  31-89 |
| **Gender** |  |  |
| Male | 190 | 170 |
| Female | 30 | 61 |
| **TNM** |  |  |
| I | 93 | 36 |
| II  III  IV  NA | 77  44  -  2 | 105  71  19  - |
| **BCLC** |  |  |
| 0  A | 20  148 | -  - |
| B  C  NA | 22  28  2 | -  -  - |
| **OS Status** |  |  |
| Alive | 136 | 189 |
| Death | 84 | 42 |
|  |  |  |
